# Effect of Nanobubble Presence on Murine Fibroblasts and Human Leukemia Cell Cultures

**DOI:** 10.1021/acs.langmuir.2c00819

**Published:** 2022-07-01

**Authors:** Karol Ulatowski, Kamil Wierzchowski, Julia Fiuk, Paweł Sobieszuk

**Affiliations:** Warsaw University of Technology, Faculty of Chemical and Process Engineering, Department of Biotechnology and Bioprocess Engineering, Warynskiego 1, 00-645 Warsaw, Poland

## Abstract

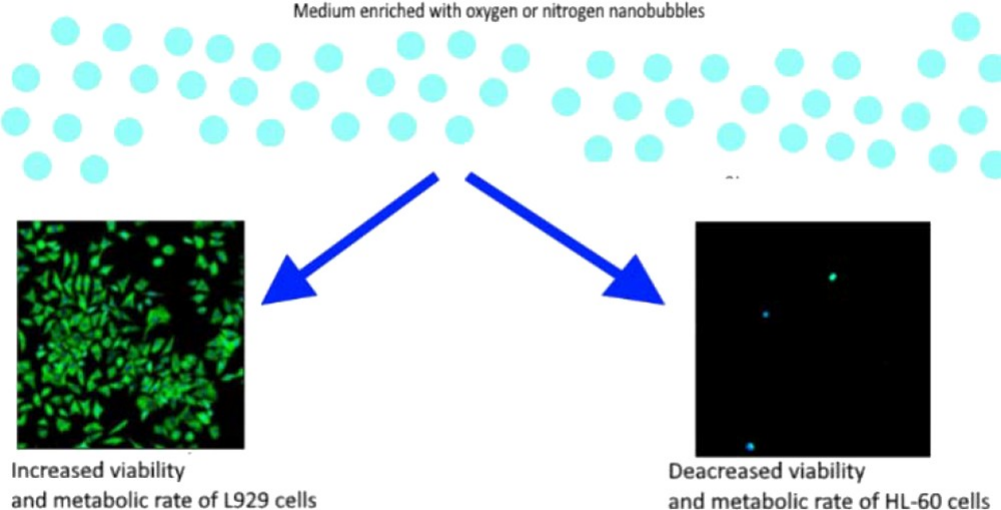

Nanobubbles can enhance both the proliferation and metabolic activity of microorganisms (mainly bacteria) and the growth of the whole higher organisms such as mice, fish, or plants. The critical fact is that nanobubbles of different gases can affect given cells differently. As animal cell cultures are used in industry and research studies, investigations of their interactions with nanobubbles should be carried out. This study aims to uncover whether the presence of nanobubbles improves the proliferation rate and metabolic activity of L929 fibroblasts and HL60 leukemia cells as exemplary animal cell lines of adherent and non-adherent cells, respectively. The long-term (8-day) cultures of both L929 and HL-60 cells with nanobubble addition to the appropriate medium were carried out. The medium was not exchanged for the whole duration of the culture. Nanobubbles of two gases – oxygen and nitrogen – were dispersed in the appropriate media and then used to culture cells. The density and viability of cells were assessed microscopically while their metabolic activity was determined using PrestoBlue or XTT assays. Additionally, we have performed the analysis of substrate consumption rate during the growth and activity of lactate dehydrogenase. We have shown that nanodispersion of both gases enhances the proliferation rate and metabolic activity of L929. For HL-60 cultures, reference cultures exhibited better viability, cell density, and metabolic activity than those with either oxygen or nitrogen nanobubbles. Obtained results clearly show that nanobubble dispersions of both oxygen and nitrogen positively affect the cultures of L929 while inhibiting the growth of HL-60 cells. We suspect that a similar positive effect would be visible for other adherent cells, similar to L929. Such results are promising for intensifying the growth of animal or human cells in routine cell cultures.

## 1 Introduction

Understanding interactions between nanoobjects and living organisms is starting to gain attention as the subject of investigation.^1−11^ Plants and animals grow larger with a micro- and nanobubble presence in the growth environment, and the reasons for such an effect are currently studied.^7,8,10−13^ Ebina et al. have shown that *Brassica campestris* plants, mice, and two fish species have grown significantly quicker with micro–nanobubbles dispersed in water than control organisms.^7^ Each organism treated with nanobubble water grew larger (higher mass and body length for animals, larger leaves, and higher mass for plants) and exhibited higher food intake. Mahasri et al. have presented their system for fish tank aeration with nanobubbles.^3^

Interestingly, the type of gas enclosed in the nanobubbles does matter. The most commonly used gases in such studies are nitrogen, oxygen, carbon dioxide, and ozone. An example of this is the intensification of germination and growth of plants, where each used gas has a different influence on different plants^12^ as some react most intensively to a nitrogen presence while others prefer watering with the highly oxygenated solution. While nitrogen was universally the most effective gas in nanobubbles for germination promotion, results varied when watering the growing plants. For example, for stem length and stem diameter of tomato plants, authors observed that oxygen nanobubbles are better for promoting growth in stem diameter while nitrogen nanobubbles cause the highest elongation rate of the stem.

Lately, the interaction of gas nanobubbles with microorganisms has gained much attention.^2,5,6,14,15^ Interactions vary from destruction of bacterial or fungal cells to promotion of growth of microorganism cultures, depending on the gas used. Ozone nanobubbles proved to be helpful in microorganism cell inactivation, even those typically known to be ozone-resistant or destroy the virus strains.^14,15^ On the other hand, contact with nanobubbles of other gases can promote microorganism cell proliferation or increase their metabolic activity. Wang et al.^9^ demonstrated that methane production is enhanced in water containing oxygen nanobubbles during the anaerobic digestion of cellulose by microorganisms from anaerobically digested sludge from the sewage treatment plant. The authors stated that the micro-oxygen environment is preferable for methane production from cellulose and that oxygen nanobubbles are sufficient to significantly increase the methane yield by increasing the rate of cellulose hydrolysis. Nanobubbles can penetrate the oligosaccharide layer thanks to their high negative surface charge and hydrophilicity, facilitating hydrogen, NAD^+^ ion, and cellulase diffusion. A higher methane yield is present due to better accessibility of the substrate for the cellulose hydrolysis step of methane production. Interestingly, Luu et al.^6^ observed that oxygen nanobubbles positively impact the cell density of *Escherichia coli* while decreasing the cell density of *Pseudomonas aeruginosa*. Additionally, when bacteria were cultured with nanobubble addition, the lag phase of *E. coli* was shorter, and the growth rate was higher in the exponential phase. More importantly, bacteria length increases when they are exposed to the nanobubbles. *E. coli* contains higher protein mass per dry mass and, despite a lower growth rate, *P. aeruginosa* contains more lipids per dry mass when cultured in bubble-rich media. Effects of nanobubble presence in water on *E. coli* bacteria were also investigated by Yamaguchi et al.^1^ Authors uncovered that oxygen nanobubble presence enhances the survival rate of bacteria in pure water. In contrast, nitrogen nanobubbles are generally neutral, and carbon dioxide nanobubbles were causing a decrease in survival rate compared to pure water. Authors attributed these effects to the presence of reactive oxygen species. Guo et al.^4^ showed that *Lactobacillus acidophilus* 1028 cells displayed a higher growth rate and lactic acid production when exposed to nanobubble-rich water. The higher lactic acid production was observed for nitrogen and hydrogen nanobubbles while a higher growth rate was observed for carbon dioxide and air nanobubbles.

Previous studies of our research team have shown that the effects of nanobubble presence are also visible in yeast cultures. We used nanobubbles in the *Saccharomyces cerevisiae* yeast culture in 3 and 5 L vessels.^5^ Based on the elemental balance of growth, we deduced that the oxygen enclosed in nanobubbles would not be sufficient to maintain the culture for the typical concentration of nanobubbles generated using the hydrodynamic method. We have carried out a typical batch culture with barbotage aeration, but the inoculum and broth were saturated with oxygen nanobubbles. Additionally, we carried out sequential batch cultures (where part of the culture volume was exchanged with fresh nanobubble-saturated medium) and semi-batch cultures (where the fresh medium was continuously supplied to the culture vessel). We showed that even the presaturation of culture with nanobubbles allowed for the higher growth rate of yeast with higher substrate consumption and a shorter adaptation (lag) phase. Comparison between batch, sequential, and semi-batch cultures shows that the more continuous the supply of nanobubble-saturated broth, the higher the maximum specific growth rate of yeast. This parameter was defined using Monod and Tsao–Hanson models. The collective findings indicate that contact with a dispersion of nanobubbles of nontoxic gases increases the metabolic rate of microorganisms.^4−6,9^ Guo et al. also suggested that the gas used to generate nanobubbles impacts the bacteria, but more importantly, the sole presence of gas nanobubbles of any gas, even those considered neutral, influences the growth and metabolic rate of bacteria.^4^

Results reported in the literature for bacteria and yeast cultures are auspicious from both scientific and industrial points of view as numerous processes are based on microorganism usage, such as organic acid production, wastewater treatment, microalgae cultures for photosynthesis, and organic oil production, among others. However, in the last few decades, the processes involving cultures of cells of higher organisms are emerging, such as artificial meat production, culturing for monoclonal antibodies, and others. That means that the studies of nanobubble–cell interactions are an essential subject to pursue. As animal cell cultures are the tool used in both industry and research studies, their interactions with nanobubbles are an interesting next step in research studies, especially since the effect on whole organisms is already proven, as mentioned earlier. In this work, we aim to uncover how the nanobubble dispersions of nitrogen and oxygen are affecting the proliferation rate, viability, metabolic activity, and substrate consumption rate during long-term cultures of two animal cell lines: murine fibroblast cell line (L929) and human leukemia cell line (HL-60) as the model cell lines of adherent and non-adherent cells, respectively. That allows determining whether the cells growing in the bulk of liquid react differently to the nanobubble stimuli than those growing in the monolayer attached to the vessel’s surface. Investigation of both nitrogen and oxygen nanobubbles in cultures with both of these cell lines allows us to distinguish whether the effect is connected to the sole presence of inert nanobubbles (in the case of nitrogen nanodispersion) or the higher and long-lasting concentration of oxygen coupled with nanobubble presence (oxygen nanodispersion).

## 2 Materials and Methods

### 2.1 Preparation of Nanobubble-Enriched Culture Media

**Figure 1:**
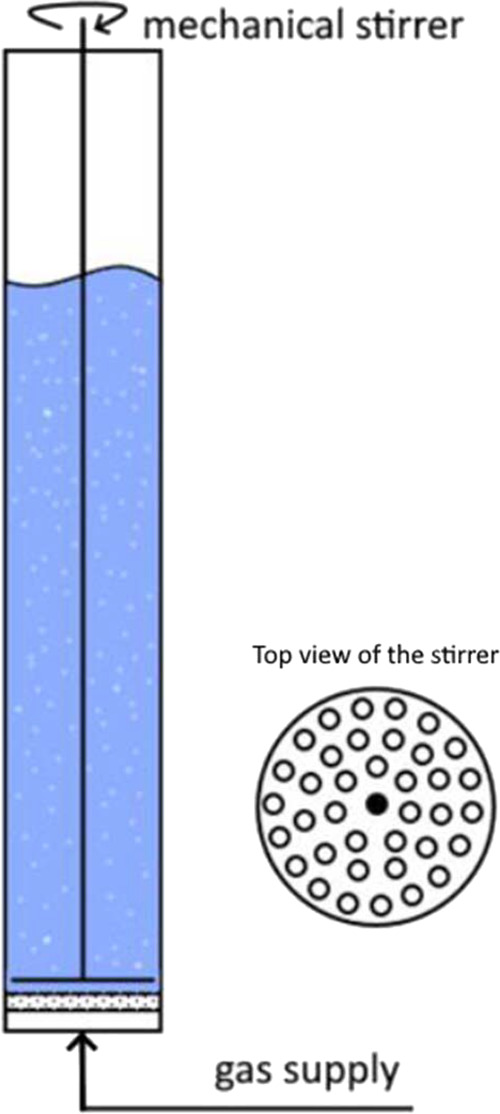
Schematic view of the generation setup.

Nitrogen or oxygen gas nanobubble dispersions were prepared using the PVC hollow cylinder (internal diameter 25 mm) with the round silicon carbide membrane (pore diameter of 0.2 μm; Cembrane, Norway) embedded in the cylinder. The schematic drawing of the setup is presented in Figure 1.

The cylinder was filled with 50 mL of appropriate culture medium, either DMEM (for L929 cell cultures) or RPMI (for HL-60 cultures), and the mechanical high-shear stirrer was set 1 mm over the membrane. Gas was supplied from the nitrogen or oxygen cylinder (gas purity 99.99%, Air Products, Poland) with pressure under 0.1 bar. The impeller rotation rate was set at 900 rpm. Due to shear stress induced on the membrane surface, the nanobubbles were cut off from the membrane surface and transferred to the bulk of the liquid. After 20 min of generation, the gas flow was stopped, and freshly generated nanodispersion was filtered using a syringe pump and Fisherbrand Sterile PES (φ = 0.2 μm) membrane filters, as described in detail in our previous work.^16^ Nanobubble dispersions were used the same day. A basic stability assessment was performed by measuring the size of nanobubbles in blank samples with nanodispersion during storage using a Zetasizer NanoZS (Malvern Panalytical, United Kingdom). Additionally, the oxygen concentration assessment was carried out for oxygen nanodispersions in media to ensure that the nanobubbles were present in blank samples after 8 days of culture, using ProSolo (YSI) optical sensor. Samples denoted as “blank” were not mixed with the cell suspension. Their purpose was to determine whether nanobubbles can be preserved in culture conditions. The “blank” descriptor was added to distinguish them easily from the reference samples gathered from cultures without nanobubble addition.

### 2.2 Preparation of Cell Suspensions

#### 2.2.1 L929 Cell Line

L929 cells were cultured in DMEM medium (glucose, 1 g/L; l-glutamine, 4 mM; 10% FBS; 1% Pen-Strep; pyruvate, 1 mM; without phenol red; Gibco) with cell passage each 2–3 days. When cells reached the exponential growth phase, they were detached from the vessel’s surface. Wierzchowski and Pilarek^17^ described the procedure of adherent cell detachment that was applied in this study. Briefly, the method was carried out in the following steps. The culture medium was pipetted out, and still adhered cells were flushed twice with fresh DPBS without calcium or magnesium ions. Next, cells were detached by adding trypsin–EDTA and leaving them for 5 min at room temperature to accomplish trypsinization. Then, DMEM culture medium was added, and cells were suspended by gently shaking the culture flask. Next, maintained cells were separated from the culture medium and suspended in freshly generated dispersion of either oxygen or nitrogen nanobubbles in DMEM or pure DMEM medium to achieve the concentration of 1 × 10^5^ cells/mL. Media with cells were mixed with fetal bovine serum (FBS; Gibco) and a Pen-Strep antibiotic mixture (Gibco) in volume proportion DMEM/FBS/Pen-Strep of 89/10/1.

#### 2.2.2 HL-60 Cell Line

HL-60 cells were cultured in the RPMI (glucose, 2.0 g/L; l-glutamine, 4 mM; supplemented with 10% FBS; 1% Pen-Strep; pyruvate, 1 mM; without phenol red; Gibco) with the passage of cells each 2–3 days. Due to cell growth in the bulk of the liquid, detachment from the surface was not necessary. After the initial culture, maintained cells were separated from the culture medium and suspended in freshly generated dispersion of either oxygen or nitrogen nanobubbles in RPMI and pure RPMI medium to achieve the concentration of 10^5^ cells/mL (for cell cultures) or 5 × 10^5^ cells/mL (for cytotoxicity tests). The difference between these cell number densities comes from the fact that for cytotoxicity tests, we often start with the more dense culture of cells as it allows for a higher response in the cytotoxicity test, which helps achieve larger differences between samples with similar metabolic activities. For that reason, in preliminary studies described before long-term cultures, higher cell densities were used. In the case of cell cultures, one has to leave cells room to grow, and therefore, for cultures, the initial densities were 5 times smaller. Of course, all metabolic activity tests carried out during the culture, including tests similar to those used in preliminary studies, were also carried out for lower cell density. That allows us to be certain that all cells grow and proliferate in the same conditions. Media with cells were mixed with fetal bovine serum (FBS) and a Pen-Strep antibiotic mixture in volume proportion RPMI/FBS/Pen-Strep of 89/10/1.

### 2.3 Cytotoxicity Tests

#### 2.3.1 XTT Tests for L929 Cell Line

The cell suspension was added to 96-well plates (100 μL/well) and incubated at 37^°^C, 5 % CO_2_ for 24 h. Each plate also contained blank references (wells without cells but with investigated dilutions), negative control (wells containing cells with non-diluted DMEM medium), and positive control (cells with DMEM medium with 0.1% TritonX added). For each experiment, five 96-well plates were used. Six replicate wells were used for each sample on each plate, i.e., for each metabolic test or viability assessment. Then, the XTT test was carried out. 50 μL of test mixture (CyQUANT XTT Cell Viability Assay, ThermoFisher Scientific) was added to each well, and plates were incubated in darkness at 37^°^C, 5 % CO_2_ for the next 4 h. After that time, the absorbance of solutions at 470 nm (with a control wavelength of 650 nm) was measured using a spectrophotometer against blank references. The activity of the negative control sample was assumed as 100% metabolic activity of cells, and metabolic activities in other samples were compared to that value.

#### 2.3.2 Presto Blue Tests for HL-60 Cell Line

Cell suspensions were sampled to a 96-well plate (100 μL/well). For each experiment, five 96-well plates were used. Six replicate wells were used for each sample on each plate, i.e., for each metabolic test or viability assessment. After 24 h of culture, the Presto Blue assay was added (11 μL/well) to 100 μL of the cell suspension to obtain the concentration of PrestoBlue reagent of 10%. Plates were incubated at 37^°^C, 5 % CO_2_ for 2 h. After the elapsed time, absorbance at 570 nm with 600 nm as a reference wavelength was measured. Results were compared to the negative control, which was assumed as 100% of the metabolic activity of cells.

### 2.4 Cell Cultures

#### 2.4.1 L929 Cell Culture

After the preparation of suspensions (Section 2.2.1), cultures of L929 cells in prepared suspensions were carried out in 24-well plates (1.50 mL/well). Media samples with cells were gathered for metabolic activity and viability studies every 2 days. The first sample for visualization under confocal microscopy was gathered after 4 h of culture.

L929 cell cultures were carried out for 8 days without exchange of the medium. Each even day (0, 2, 4, 6, and 8 days after the start of the culture), the following tests or measurements were carried out: cell density and viability assessment (trypan blue dye), metabolic activity (Presto Blue assay), the activity of lactate dehydrogenase (LDH assay) and glucose concentration. Additionally, confocal microscopy was used to visualize the morphology of L929 cells in the culture at each time point.

#### 2.4.2 HL-60 Cell Culture

After preparation of the cell suspension (2.2.2), HL-60 cells were added to 24-well plates (1.50 mL/well). Media samples with cells were gathered for metabolic activity and viability studies every 2 days. Cultures lasted for up to 8 days.

### 2.5 Assessment of Properties of Cell Cultures

#### 2.5.1 Measurement of Cell Density and Viability

Cell density and viability were determined by counting cells after staining with 0.4% trypan blue aqueous solution (Thermo Fischer Scientific, US) mixed in a 1:1 proportion. A Bürker-Türk hemocytometer (Brand, DE) and an Eclipse TS100 inverted microscope (Nikon) were used to ensure the replicability of the counting. Samples of cell suspensions were used for the determination of the values of cell density, *X*, and viability of cells, *Z*, which were calculated according to eqs 1 and 2:
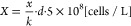
1
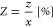
2where *x* is the total number of cells (i.e., a sum of both stained and unstained ones) counted in the grid of the hemocytometer, *z* is the number of living (i.e., unstained) cells, *k* is the number of grid squares with cells, and *d* is the dilution of the sample containing cells. Factor 5 × 10^5^ is determined by the medium’s volume over the hemocytometer’s grid.

#### 2.5.2 Metabolic Activity Assessment

Metabolic activity has been estimated by the resazurin-based PrestoBlue assay (Presto-Blue, Thermo Fischer Scientific, US). 0.156 mL of PrestoBlue reagent was added to 1.5 mL of either HL-60 culture, L929 culture, or pure culture medium without cells (in the case of reference samples), obtaining a final concentration of PrestoBlue reagent of 10%. Next, all samples were incubated for 2 h at 37^°^C. After the elapsed time, absorbance was measured using a GENESYS 20 UV–VIS spectrophotometer (Thermo Fisher Scientific, US) at 570 nm and a reference wavelength of 600 nm. Finally, the values of *a_m_* were calculated using the relations presented as eqs 3 and 4:
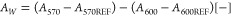
3
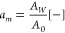
4where *A_w_* is the specific absorbance of the test sample, *A*_570_ is the absorbance of the test sample at 570 nm, *A*_570REF_ is the absorbance of the reference sample at 570 nm, *A*_600_ is the absorbance of the test sample at 600 nm, *A*_600REF_ is the absorbance of the reference sample at 600 nm, *a_m_* is the metabolic activity of cells, and *A*_0_ is the specific absorbance of the sample taken from the culture in water without nanobubbles at the start of the culture.

To better compare the results obtained for different samples in each time point, and as such, for probable different cell densities, we decided to introduce the normalized metabolic activity, *a*_*m*/*X*_ as follows:
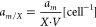
5where *a_m_* and *X* are the metabolic activity and cell density in a given time point and *V* is the volume of the culture in one well, i.e., 1.5 mL.

#### 2.5.3 LDH Activity Measurement

LDH activity has been determined according to the procedure of BioMaxima-LDH enzymatic assay (BioMaxima, PL). 1.0 mL of Biomaxima-LDH reagent was added to 20 μL of filtered (syringe filters, ϕ = 0.2 μm) culture medium sampled from cultures or mixed with 20 μL of double-distilled water (in the case of blank samples). Absorbances of such reaction mixtures were spectrophotometrically monitored in 1 min intervals using a GENESYS 20 UV–VIS spectrophotometer (Thermo Fisher Scientific, US) at 340 nm. Finally, values of LDH activity were estimated based on the following correlation, proposed by the assays’ manufacturer:
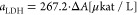
6where *a*_LDH_ is the lactate dehydrogenase activity and Δ*A* is the absorbance change per minute. Afterward, this activity was normalized using cell density to show the leakage of LDH per cell, *a*_LDH/*X*_ as follows:
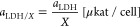
7

#### 2.5.4 Glucose Concentration Measurement and Glucose Consumption Rate Calculation

Glucose consumption rate has been estimated based on the monitored glucose level in culture medium by the BioMaxima-glucose enzymatic assay (BioMaxima, PL). 1.0 mL of BioMaxima reagent was mixed with 20 μL of filtered (syringe filters, ϕ = 0.2 μm) culture medium gathered from the cultures (in the case of the test sample) or with 20 μL of double-distilled water (in the case of the blank sample). Next, all samples were incubated for 20 min at room temperature before absorbance measurements using a GENESYS 20 UV–VIS spectrophotometer (Thermo Fisher Scientific, US) at 550 nm. Based on absorbance, the glucose concentrations were calculated. Finally, values of the glucose consumption rate normalized by the average concentration of glucose were determined according to the following equation:
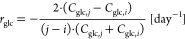
8where *r*_glc_ is the glucose consumption rate and *C*_glc, *i*_ and *C*_glc, *j*_ are the glucose concentrations in the culture medium at time stamps *i* and *j* (for *j* > *i*).

#### 2.5.5 Morphology and Cell Culture Visualization

L929 cells were maintained in DMEM medium with or without nanobubble addition on round slides (diameter 13 mm, thickness 0.3 mm, Bionovo, PL) placed in the 24-well plates. After 0, 2, 4, 6, and 8 days of culture, part of the slides with cells was transferred to new 24-well plates and washed twice with DPBS without calcium and magnesium ions. Next, collected slides with cells were soaked in the following solutions: 1 mL of 4% (w/v) paraformaldehyde in water solution (PFA, Sigma-Aldrich), 1 mL of 0.2% (v/v) TritonX-100 in water solution (Sigma-Aldrich), 200 μL of 165 nM Alexa Fluor 488 Phalloidin (Thermo Fisher Scientific) in DPBS solution, and 100 μL of 300 nM DAPI (Thermo Fisher Scientific) in DPBS solution; and after each step, the slides with cells were washed twice with 1 mL of DPBS without calcium and magnesium ions. Samples were visualized by an LSM 880 confocal laser scanning microscope (Carl Zeiss AG, Jena, Germany). A similar procedure is impossible for HL-60 cells because these cells are non-adherent and would not be preserved on the glass slide.

## 3 Results and Discussion

### 3.1 Properties of Nanobubble-Enriched Media

To validate whether studies of long-term cultures are viable, we have assessed the stability of the size of nanobubbles and oxygen concentration in the liquid in blank samples stored separately in sterile conditions. Table 1 presents the simple assessment of the size stability of nanobubbles in blank samples, which were stored in the same conditions as the cultures with nanobubbles to check whether nanobubbles remain in culture during its whole length. During storage, size values fluctuated for both media and gases enclosed in nanodispersion, but the size of bubbles after 1 day (24 h) was similar to the value after 8 days (168 h) of storage.

**Table 1:** Sizes of Nanobubbles in Blank Samples of Nanodispersions in Media in Time

	oxygen nanodispersion		nitrogen nanodispersion	
time [h]	DMEM	RPMI	DMEM	RPMI
<1	192 nm ± 67 nm	174 nm ± 54 nm	221 nm ± 65 nm	204 nm ± 48 nm
24	245 nm ± 82 nm	492 nm ± 31 nm	355 nm ± 53 nm	434 nm ± 43 nm
168	170 nm ± 10 nm	560 nm ± 78 nm	312 nm ± 27 nm	373 nm ± 52 nm

In blank samples measured using an optical probe, oxygen concentrations were 312% and 289% of the equilibrium concentration with air for DMEM and RPMI, respectively. This value quickly decreased because of gas desorption from the liquid but stayed at about 7% higher than equilibrium concentration with air for 8 days for both DMEM and RPMI as the continuous phase of the nanodispersion.

This experiment showed that nanobubbles were present in nanodispersion, and their size stabilized on specific values depending on the medium and gas used. Additionally, the oxygen concentration remained higher than in the case of reference samples. Based on the analysis of the stability of the size of nanobubbles, we showed that nanobubbles are present in the dispersion during the whole experiment duration. For that reason, we expect that nanobubbles were able to affect the culture for a whole 8 days during long-term cultures.

One has to note that this is only the assumption because we are not able to measure the density of nanobubble size distribution in the culture. The measurement of nanobubble size during cell culture is impossible due to two main reasons: (i) the exact rheological and optical parameters of dispersion during culture will change due to excretion of metabolites, which deems DLS measurements unreliable; (ii) in the case of non-adherent cell culture, to separate the cells from the culture medium, one has to centrifuge the cells, and that process will destroy or separate also nanobubbles from medium due to the difference in density. On the other hand, the excretion of metabolites will probably change the surface charge of nanobubbles, which may affect their size and stability. Because of that fact, the most crucial proof of nanobubble existence is differences between reference cultures and cultures with nanobubble additions. Such differences are observed and will be discussed in the following sections.

### 3.2 Effect of Nanobubble Presence in Medium on Metabolic Activity, Viability, and Substrate Consumption Rate during Long-Term Culture

#### 3.2.1 Cytotoxicity Tests

Cytotoxicity tests of nanobubbles generated directly in a culture medium (DMEM for L929 cells and RPMI for HL-60 cells) were carried out to determine the primary, short-term effect of the generation of nanobubbles directly in the medium.

**Figure 2:**
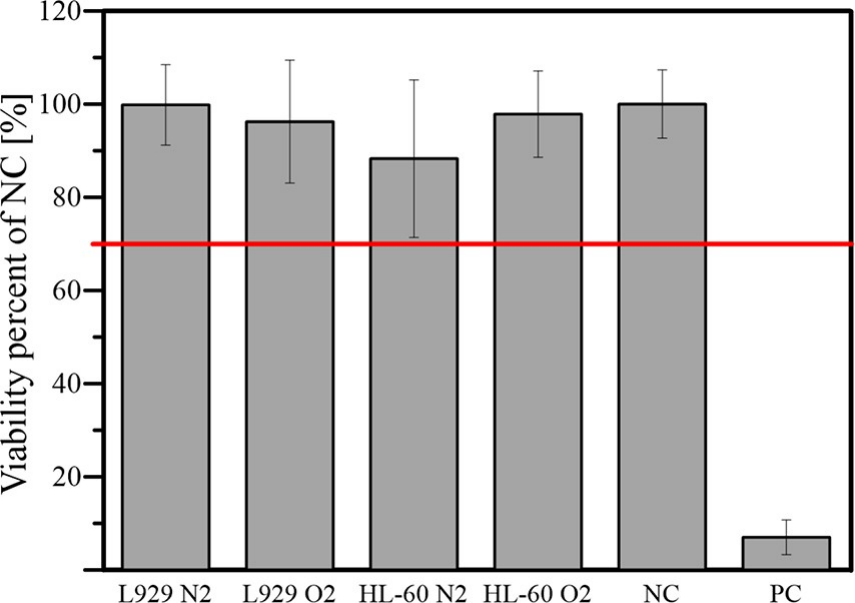
Cytotoxic effects of nitrogen and oxygen nanobubbles dispersed directly in media on L929 and HL-60 cells after 24 h incubation with nanobubble dispersion of either oxygen (samples denoted O2) or nitrogen (samples denoted N2) as a percent of negative control viability. The horizontal line shows the generally accepted boundary of cytotoxicity at 70% of the activity of negative control (NC). Positive control was cell cultures with TritonX-100. The differences between negative control and cultures with nanobubble dispersions are not statistically significant for α = 0.05, according to the post-hoc Tukey test.

Figure 2 presents the results of the XTT (for L929) and Presto Blue (for HL-60) cytotoxicity tests. One can see that all of the tests showed that nanobubbles are not cytotoxic for cells as the metabolic activity is not below 70%, which is commonly set as the boundary of cytotoxicity. For nanobubbles of nitrogen generated in RPMI, the response of HL-60 cells bears the most significant standard deviation of results. The average value is much lower than oxygen nanodispersion in the RPMI medium or dispersions of both gases affecting L929 cells in the DMEM medium, but the differences are not statistically significant based on the post-hoc Tukey test. The cytotoxicity tests indicated that neither media with nanobubbles are toxic for L929 or HL-60 cells. As such, the following studies were justified.

#### 3.2.2 Long-Term Cultures of L929 Cells

##### 3.2.2.1 Morphology Assessment

**Figure 3:**
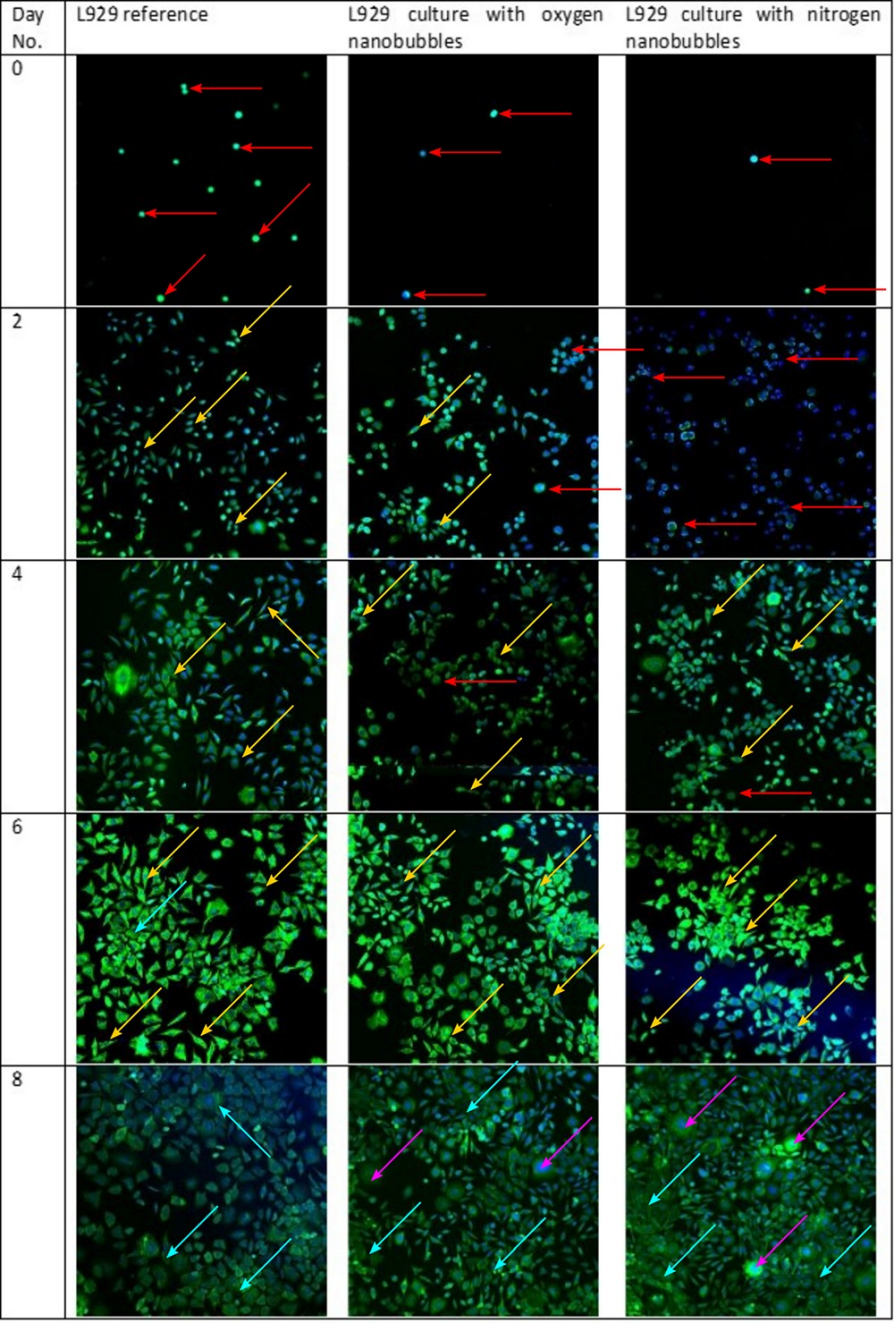
Morphology assessment of L929 cells using confocal microscopy. The arrows point to cells with different morphologies: red arrows – round, not-adhered cells; yellow arrows – spindle-like cells; light blue arrows – cobblestone-like cells; purple arrows – multinucleated, senescent cells.

Photographs of cells after staining are presented in Figure 3. The actin cytoskeleton of the cells is stained green while nuclei are stained blue. Different morphologies of cells were indicated using different colors of arrows, i.e., red for round-shaped, yellow for spindle-like-shaped, light blue for cobblestone-like-shaped, and purple for multinucleated cells.

One can see a difference in cell density between reference cultures and cultures with nanobubbles after 4 h of culture, suggesting that nanobubbles decrease the adhesion rate of L929 cells. After 2 days of culture, the cells in the reference culture properly adhere to the slide surface and have typical, spindle-like morphology. Cells cultured in medium with nitrogen nanodispersion had primarily round shapes, but we can see multiple cells elongated with visible necking, suggesting that cells were in the middle of the division. Another fact confirming this hypothesis is that cell nuclei are fused with the cytoplasm (the green actin cytoskeleton is not visible, only blue nuclei), which is a step during mitotic cell division. It may suggest that the nanodispersion presence influences cell proliferation. In the case of oxygen nanodispersion, most cells have a spindle-like shape, but some are round, and few also have visible necking, similar to those cultured with nitrogen nanobubble dispersion. That shows that the adhesion of cells in culture with added nanodispersion is slower than in reference. Cells may pursue different metabolic pathways, probably connected with their proliferation. After 4 days of culture, the morphology of cells in reference cultures and those with oxygen nanodispersion has not changed from day 2. However, in the case of nitrogen nanodispersion, more cells achieved typical, i.e., spindle-like, morphology. After 6 days, cells were mainly spindle-like in all of the cultures. Curiously, the nuclei of cells after 6 days are not as clearly visible as in the previous days, especially for culture with nanobubble addition. After 8 days of the culture, we can see vast agglomerates of cells covering nearly the whole surface of the slide – the over confluent culture. In agglomerates, the cell morphology changed from spindle-like to cobblestone-like. Also, some cells are much larger than the surrounding ones and are multinucleated, which points to senescence and growth without cytokinesis, as described in the literature.^18^ The highest number of such cells is visible in the culture with nitrogen nanobubble addition, which shows that the cells underwent senescence faster after the first drastic increase of proliferation rate and multiple simultaneous divisions.

##### 3.2.2.2 Culture Condition Assessment

**Figure 4:**
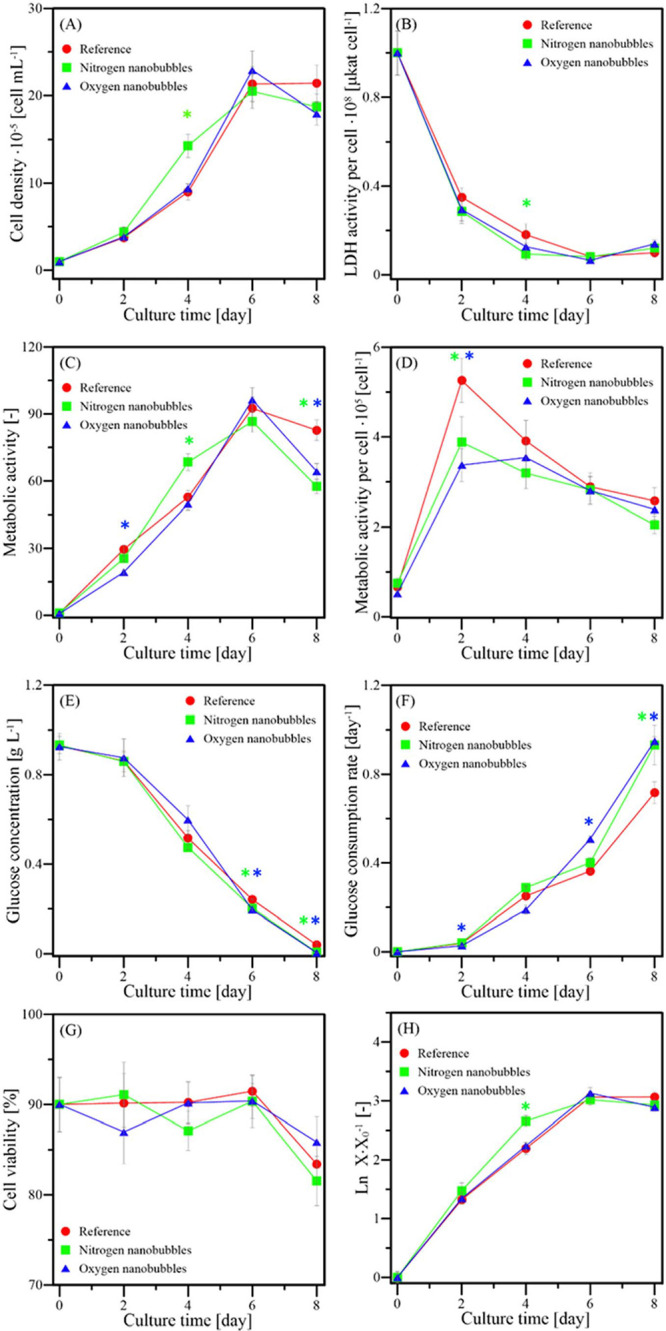
L929 culture properties at different time points. (A) cell density, (B) LDH, (C) cells’ metabolic activity evaluated using PrestoBlue, (D) normalized cells’ metabolic activity evaluated using PrestoBlue, (E) glucose concentration, (F) glucose consumption rate, (G) viability of cells, and (H) logarithmic ratio of cell density to initial cell density. The asterisks mark the time points at which the culture with nitrogen (green asterisk) or oxygen (blue asterisk) nanodispersion was statistically different from the reference culture with α < 0.1 according to the post-hoc Tukey test.

Figure 4 presents the results for L929 cultures, showing how different culture parameters changed for different samples. Figure 4A shows the cell densities measured for all three types of samples. The typical growth curve was obtained for both nanodispersions in medium and reference samples, where the adaptation (lag) phase is extremely short, the exponential growth phase lasts from 0th to about 6th day for culture with oxygen nanodispersion and the reference culture while for the nitrogen culture, it lasted from 0th to 4th day. Both stationary and decline (death) phases happen between the 6th and 8th day (reference and oxygen nanodispersion cultures) or between the 4th and 8th day (nitrogen nanodispersion culture). The above characteristics of the growth curve are further shown in Figure 4H. As mentioned earlier, cells’ death and senescence are also visible in Figure 3, which confirms the death phase’s existence. The specific growth rate in the reference sample corresponds to literature results^19,20^ and is presented along with specific growth rates in samples with nanodispersion addition in Table 2.

**Table 2:** Specific Growth Rates of L929 Cells in Different Media

L929 ref. [1/day]	L929N2 [1/day]	L929 O2 [1/day]
0.510	0.665	0.522

For nitrogen nanodispersion, the cell density after 4 days of culture was significantly higher than in the case of oxygen nanodispersion or reference samples. All samples had similar cell density after 2 days of culture, clearly showing a higher proliferation rate obtained for nitrogen nanodispersion between the 2nd and 4th day of culture. It corresponds to confocal microscopy results where cells cultured with nitrogen nanodispersion proliferated faster, and their division cycles were more synchronized. It is worth comparing these results to plots visualized in Figure 4E, where after 4 days of culture, the lowest concentration of glucose was observed for nitrogen nanodispersion. That shows that cells increased their substrate consumption to accommodate for a higher proliferation rate.

Interestingly, a similar effect is visible for oxygen nanodispersion after 6 days of culture (see Figure 4A,E). For both of these dispersions, the consumption rate in a given day normalized by the average amount of substrate between two measurements (Figure 4F) is the highest for respective nanodispersions and appropriate time points. For both nanodispersions, after 8 days of culture, we see a decrease in cell density, which shows that cell deaths were more frequent than cell divisions. That corresponds to the results obtained using confocal microscopy, where multiple dead cells were visible after 8 days of culture. The apparent reason is the extremely low concentration of glucose, which is the primary nutrient for cells in culture and is necessary to maintain cell life. The viability of cells was nearly constant, with all three types of culture maintaining 90 % ± 5% for the first six days of culture, decreasing only after 8 days to the minimum of 81 % ± 2% (see Figure 4G).

The presented results correspond to the above literature studies, claiming that murine fibroblast cells prefer the hypoxic environment for proliferation.^21^ The dissolved oxygen is purged from the medium during the preparation of nitrogen nanodispersion. In that case, the oxygen concentration is lower and closer to the physiological concentration of oxygen (∼5%).

Analysis of the metabolic activity (Figure 4C) shows a good approximation of growth curves (Figure 4A). After 2 and 8 days of culture, reference samples exhibited the highest activity while after 4 days, the activity of reference culture was lower than for the one cultured with nitrogen nanodispersion, which is confirmed by the post-hoc Tukey test. The slightly higher reference culture value than the one cultured with oxygen nanodispersion bears lower statistical significance. Similarly, L929 cells cultured with oxygen nanodispersion show the lowest metabolic activity after 2 and 4 days while having the highest activity after 6 days of culture. Cell density in a medium with nitrogen nanodispersion seems to show the opposite effect, being in the lead after 4 days of culture and falling behind in the next 4 days. However, when we calculate the average metabolic activity of a single cell by normalization using cell density in respective samples, we observe that some of these effects are turned upside down while others are even more visible. Activity per cell for the reference sample (Figure 4D) after 2 days of culture is much higher than it would seem from the analysis of Figure 4C as the cell density was the lowest. We see that despite slower growth, metabolic activity in the sample without nanobubbles was the highest in the average cell. After 4 days of culture, thanks to the highest proliferation rate, the nitrogen nanodispersion showed much higher metabolic activity of cells (Figure 4C) but the lowest activity per cell (Figure 4D). The cost of the fastest proliferation rate was significantly decreased mitochondrial activity of individual cells. Senescence of L929 cells after 8 days of culture, causing the emergence of multinuclear cells (as visible in Figure 3), may also cause the lowest activity of cells cultured with nitrogen nanobubbles, where the senescent cells were most common.

Figure 4B shows the lactate dehydrogenase (LDH) activity, which measures cell damage that causes the secretion of this enzyme to the cell exterior. LDH activity in reference culture corresponds to previous studies.^20,22^ For that reason, the higher LDH concentration in the medium per cell means that many cells have been recently damaged. It is complementary to analyzing cell density and viability in assessing culture conditions. After 2 and 4 days of culture, one can observe the highest value for reference culture, which shows that despite the highest mitochondrial activity, the cells are struggling to stay undamaged compared to samples with nanodispersion. After 8 days of culture, the LDH activity for all samples is significantly increased, corresponding to viability and cell density results.

#### 3.2.3 Long-Term Cultures of HL-60

**Figure 5:**
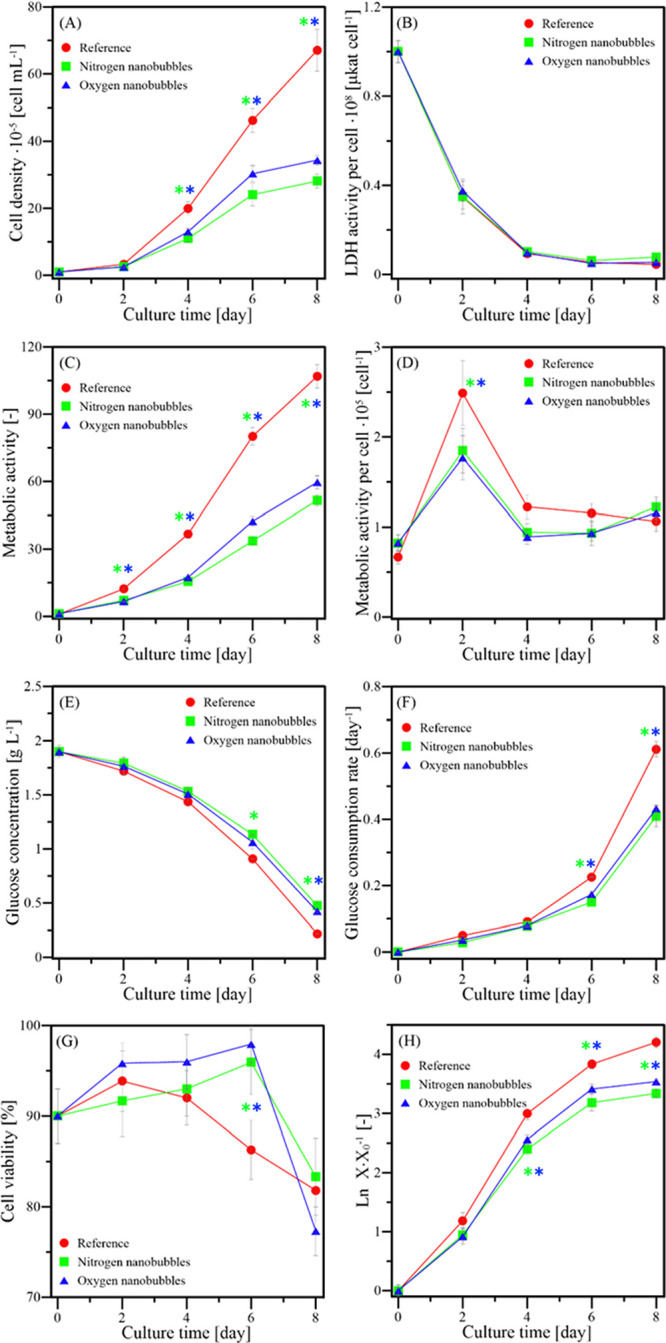
HL-60 culture properties at different time points. (A) cell density, (B) LDH, (C) cells’ metabolic activity evaluated using PrestoBlue, (D) normalized cells’ metabolic activity evaluated using PrestoBlue, (E) glucose concentration, (F) glucose consumption rate, (G) viability of cells, and (H) logarithmic ratio of cell density to initial cell density. The asterisks mark the time points at which the culture with nitrogen (green asterisk) or oxygen (blue asterisk) nanodispersion was statistically different from the reference culture with α < 0.05, according to the post-hoc Tukey test.

As one can see in Figure 5A,H, the growth of HL-60 cells was exponential from day 0 and lasted till day 6. Somewhere between the 6th and 8th day, the stationary phase started. It is the main difference between adherent cells (like L929) and non-adherent cells (like HL-60). The adaptation phase is nearly non-existent due to the lack of the necessity of adhesion to the vessel’s bottom. As for the comparison between samples, the cell density for reference culture is much higher than for samples with nanodispersion while the oxygen nanodispersion allowed for a higher growth rate than nitrogen nanodispersion for the whole culture duration. No samples exhibited a decrease in the cell density, which would signify highly unfavorable conditions for HL-60 cells. It is expected in non-adherent culture as HL-60 cells can grow suspended in the bulk of the liquid, and contact inhibition effects do not highly limit the growth. It is worth noting that after 4 days of culture with either nanodispersion, the growth slows down, and the stationary phase after exponential growth starts to form. It may be caused by the flotation effect or surrounding the cells with nanobubbles, which slows down the mass transfer from and to the cells. Flotation is often observed when a mixture of nanobubbles and microbubbles increases the buoyancy of objects dispersed or suspended in the liquid.^23,24^ However, the nanobubbles themselves, after separation from microbubbles, are also believed to be viable for improvement of flotation processes^25^ although nanobubbles are not floating to the surface thanks to their diminutive size and nearly non-existent rising velocity. However, as proven by multiple research,^26−28^ when bubbles (of all sizes) encounter contamination in liquid to which they can adhere, they either carry it to the surface one by one by themselves or multiple bubbles adhere to the single contamination and they effectively reduce the overall density of contamination enough for it to be floated to the surface or at least closer to it. The first case occurs for the bubbles larger than contamination (macrobubble flotation) while the latter is for bubbles much smaller than contamination particles (micro-/nanobubble flotation). In our opinion, there is no reason the mechanism of flotation of cells would be different from the flotation of inorganic contaminants – bubbles can adhere to the cells and suspend them near the free surface of the liquid. Such an effect is not probable in the case of adherent cells that are firmly attached to the vessel’s surface. If that is the case, the HL-60 cells may be possibly floated to the free surface. If they remain there, they may be much closer together than in the bulk of liquid, causing them to experience contact inhibition effects, significantly slowing down their growth. On the other hand, if the nanobubbles surround the cells and do not cause them to be floated to the surface, they may still hinder the mass transfer to or from the cells by forming the layer of gas bubbles. Mentioned effects would not be possible for the adherent cells, immobile and firmly linked to the vessel’s surface. As HL-60 cells grow in the bulk of the liquid and can move freely, they have a higher probability of encountering the nanobubbles dispersed in liquid than L929 cells, which are adhered to the bottom of the culture vessel. The HL-60 cell should have a higher percentage of its surface covered in nanobubbles than the L929 cell. It may mean that for L929, where the coverage by nanobubbles is visible but not high, the positive effect of nanobubble presence occurs while for HL-60, where the coverage is too high, the effect of hindering the mass transfer overcomes the positive influence of nanobubble presence.

The curious case is that for HL-60, the oxygen nanobubbles caused a higher proliferation rate than nitrogen nanobubbles, contrary to the results obtained for L929 cultures (compare Figures 5A and 4A). Oxygen accessibility for HL-60 is higher, as nanobubbles are also dispersed in the bulk. Even when bubbles cause the flotation of cells, HL-60 can still use oxygen dispersed in the liquid while also gaining access to atmospheric oxygen. That allows them to grow faster than L929 cells, which can gain oxygen only from the liquid directly above the cell monolayer. Additionally, as mentioned earlier, murine fibroblasts cells are better suited to proliferate in a state of hypoxia, which was not reported for HL-60 cells. The calculated specific growth rates in the exponential phase of growth of HL-60 cells are presented in Table 3.

**Table 3:** Specific Growth Rates of HL-60 Cells in Different Media

HL-60 ref. [1/day]	HL-60 N2 [1/day]	HL-60 O2 [1/day]
0.639	0.530	0.569

Moreover, as culture was carried out without medium exchange or substrate supply, the limiting factor can be the availability of the substrate. As the proliferation rate was higher in the case of reference samples, the glucose concentration (Figure 5E) was lower than for samples with both nanodispersions, and the substrate consumption rate (Figure 5F) was higher for each time point. Although the reference samples have consumed most of the substrate, the amount left was enough for the culture to not stop the exponential growth, but one can see that the growth has slowed down. Figure 4G shows that the viability for all cultures for the first 4 days was in the range 95 % ± 5% while in the following days, the reference culture viability (after the 6th day) or all cultures (after the 8th day) have decreased significantly. That shows that the reduction in glucose concentration caused some cells to die, but the death rate was still lower than the proliferation rate.

Figure 5C presents the results for the metabolic activity obtained using the PrestoBlue assay. What is crucial is that metabolic activity corresponds highly to the cell density (Figure 5A). After calculating the value of metabolic activity per cell (Figure 5D), most of the results range between values of 0.5 × 10^–5^ and 1.5 × · 10^–5^day^–1^, i.e., they are nearly constant. The only exception is the effect after 2 days of culture, where normalized metabolic activity is much higher for all investigated samples. It may be easily explained by the cells having the largest substrate pool and nearly no competition in acquiring it, which prepares them for rapid proliferation in the following days. Interestingly, the normalized value for reference after 8 days of culture has dropped below values for nanodispersions, which may be caused by the low substrate accessibility (glucose concentration below 0.25 g/L, see Figure 5E) or by the increase in the death rate (see Figure 4G).

As for the LDH activity per cell (Figure 5B), one can see that it is the highest at the culture start and quickly drops after cells have adapted to the environmental conditions. Values of LDH activity per cell after 2 days of culture and onward are low, and as such, one can conclude that the culture conditions are not causing significant damage to cultured cells.

## 4 Conclusions

The effect of nanobubble dispersions of nitrogen and oxygen on cell cultures of L929 and HL-60 was investigated. We have uncovered whether nanobubble dispersion has cytotoxic effects on the two cell lines’ cultures. As dispersions proved not to be cytotoxic, we decided to carry out long-term cultures of adherent (L929) and non-adherent (HL-60) cells with nanobubbles generated directly in the culture medium. Cultures lasted for 8 days without medium exchange. Reference cultures of mentioned cell lines in media without nanobubble addition were carried out. L929 cells cultured with nitrogen nanobubble addition to media had a much higher proliferation rate in the first days of culture than reference cultures, higher glucose consumption without loss in viability, or increased LDH leakage to the medium. Oxygen nanobubbles added to the L929 culture also increased the proliferation rate, albeit not as high as nitrogen nanobubbles, with higher metabolic activity and comparable viability to the reference cultures. However, different effects were visible for the non-adherent HL-60 cells, where the addition of nanobubbles has decreased the mentioned parameters of the culture – lower cell density, lower substrate consumption, and lower metabolic activity. These parameters are better for nanodispersions than reference only in the last 2 days of culture, but it is linked to a very low glucose concentration in reference cultures, which is the direct consequence of higher consumption on previous days. The described effect may be linked to the flotation by the nanobubbles, which causes non-adherent HL-60 cells to rise to the medium surface, or surrounding the cells by the nanobubbles, which hinders the mass transfer of both substrates and metabolites. Flotation does not occur for adherent L929 cells. Observed differences in nanobubble influence on adherent and non-adherent cells are interesting. However, in our opinion, the mentioned mechanisms are most probably not the only ones having an effect on these phenomena. The investigation of possible flotation and hindering of mass transfer by nanobubbles is the subject worth pursuing, and it requires further studies.

Investigations presented in this study show that nanobubbles in medium directly influence the cell density and metabolic activity of L929 and HL-60 cells. Results are complementary to experiments reported in the literature, carried out for microfungi and bacteria, which show the change of metabolic activity in contact with nanobubbles. Increased proliferation of adherent cells and a higher metabolic rate may be crucial in many fields, such as tissue regeneration, metabolite, and artificial meat production, but they require further studies and carrying out these processes with nanobubble addition.
